# Potential pest transfer mediated by international ornamental plant trade

**DOI:** 10.1038/srep25896

**Published:** 2016-05-25

**Authors:** Jiří Patoka, Martin Bláha, Lukáš Kalous, Vladimír Vrabec, Miloš Buřič, Antonín Kouba

**Affiliations:** 1 Department of Zoology and Fisheries, Faculty of Agrobiology, Food and Natural Resources, Czech University of Life Sciences Prague, Prague, Czech Republic; 2 Faculty of Fisheries and Protection of Waters, South Bohemian Research Center of Aquaculture and Biodiversity of Hydrocenoses and Research Institute of Fish Culture and Hydrobiology, University of South Bohemia in České Budějovice, Vodňany, Czech Republic

## Abstract

In recent years, the keeping of ornamental freshwater animals and plants in garden ponds has been growing in popularity. Water hyacinth (*Eichhornia crassipes*) is one of the preferred macrophytes seasonally imported mainly from South-eastern Asia throughout the world. This constitutes a secondary introduction inasmuch as the species is native to South America. Although many assemblages of aquatic invertebrates have been described as associated with this plant in the wild, there has been no research focused on their potential introduction via the international plant trade. We examined 216 specimens of water hyacinths imported for ornamental purposes from Indonesia into the Czech Republic. Numerous meio- and macroinvertebrates belonging to at least 39 species were captured. On the total number of individuals, the highest prevalence was of Tubulinea and Rotifera. Most of these were still alive and vital, including a caterpillar of the Indo–Australian invasive moth *Spodoptera litura*. Water hyacinths are usually placed into outdoor ponds immediately after import, which facilitates the release of non-target alien species. The present paper aims to draw attention to “hitchhikers” associated with the ornamental trade.

Biological invasions and the related homogenization of biodiversity are among the most prominent topics of biological conservation worldwide. Governments of impacted countries have spent substantial sums of money on eradicating invasive species and mitigating the damages caused by these organisms^1^,^2^. The situation is nevertheless hopeless in many cases, and effective eradication is possible only in the early stages of an invasion or if the invader occurs solely within a limited area^3^. Any information regarding possible introduction pathways for non-native species is therefore very important for conservationists, policymakers, and other stakeholders in order that they can take action in a timely manner.

The international trade in ornamental aquatic organisms is already considered one of the most important introduction pathways^2^,^4^,^5^,^6^. The attention of scientists has been drawn particularly to species kept in indoor aquaria^7^,^8^,^9^, and the risk of their introduction into the wild has been associated with the voluntary release of these animals by hobby keepers. But the risks related to species kept in ornamental outdoor reservoirs seem to be even greater because these may serve as a direct source of introduction without further human mediation^4^,^10^,^11^.

The popularity of these relatively small nature-like reservoirs (referred to hereinafter as garden ponds) has been growing in recent years and the spectrum of species advertised for keeping in said garden ponds has become gradually broader^10^,^11^,^12^. Garden ponds are usually planted with various aquatic and swamp plants and often stocked with ornamental fish, turtles, crayfish, or molluscs^13^. The risk associated with garden ponds lies in their potential link to open waters that can aid the dispersal of alien species. To date, risk studies have taken as their subjects plants and animals intentionally kept in garden ponds^14^,^15^,^16^. Although it has been presumed that the introduction of non-native invertebrates into garden ponds with ornamental plants is possible^13^, unintentionally imported organisms associated with those which are traded, known as “hitchhikers”, remain still quite understudied^17^,^18^.

Very appealing and also easy for handling and keeping are free-floating plants not penetrating the substrate (pleustophytic macrophyta). One of the most preferred ornamental pleustophytes is *Eichhornia crassipes* (Mart.), also known as water hyacinth^4^, which is native to the Amazon basin in Brazil. This neotropical perennial plant has been introduced into more than 50 countries around the world and it has become established on all continents other than Antarctica^4^,^19^.

It is a well-known phenomenon that water plants offer attractive microhabitats for various animals, and especially invertebrates^20^. The water hyacinth is no exception and associated aquatic invertebrates have been described in many regions where this plant occurs in the wild^21^,^22^,^23^,^24^.

Having in mind associated animals and the international trade, we formulated the research question of whether traded water hyacinths may serve as a transport medium for potentially invasive species.

## Results

The faunal composition more or less associated with water hyacinths in the surveyed shipment is summarized in Table 1. The most numerous groups were Tubulinea and rotifers (Rotifera), respectively comprising 53.1% and 27.6% of all identified organisms. Most prevalent were *Arcella* sp. and *Centropyxis* sp. among the Tubulinea, and *Lecane bulla* (Gosse), *L*. *leontina* (Turner), and an unidentified bdelloid species among the rotifers. Other relatively abundant groups were nematodes (Nematoda, 5.6%), oligochaete worms (Oligochaeta, 4.9%), and bryozoans (Bryozoa, 3.6%), the latter represented by statoblasts only. None of the remaining groups identified, namely gastropods (Gastropoda), planarians (Tricladida), water mites (Acari), cladocerans (Cladocera), copepods (Copepoda), ostracods (Ostracoda), springtails (Collembola), thrips (Thysanoptera), dragonflies (Odonata), true bugs (Heteroptera), flies (Diptera), hymenopterans (Hymenoptera), beetles (Coleoptera), and butterflies (Lepidoptera) comprised more than 1% of all those organisms identified (Table 1). There was no clear effect of 3 weeks incubation on number of individuals (Wilcoxon paired test: z = 0.714, P = 0.238) as same as on number of detected taxonomic groups (McNemar’s test: χ^2^ = 3.200, P = 0.074). But it must be mentioned that there was no new taxonomic group detected after rinsing and incubation, i.e. rinsing probably leads to lower diversity of “hitchhikers” and non-significant result can be a consequence of small number of samples evaluated.

## Discussion

Water hyacinths are seasonally imported as ornamental plants mainly from South-eastern Asia. This constitutes a secondary introduction inasmuch as the species is native to the Amazon basin in South America. The faunal composition of animals associated with water hyacinths from the wild has already been reported, and in particular the microhabitat of submerged roots has been highlighted as an important shelter for a rich interrhizon invertebrate community^21^,^25^. In addition to the interrhizon, however, we found some invertebrates, such as caterpillars, living above the water level, and particularly on the surface of leaves and their bulbous stalks. Moreover, some adult specimens tiny in size, such as thrips and hymenopterans belonging to the aeroplankton^26^, were found there as well. Thus, not only expected interrhizon and surprisingly not only directly associated species were transported via this introduction pathway. Even if all the aeroplanktonic animals were in this instance dead, survival in some cases cannot be reliably excluded.

Regarding Tubulinea and rotifers, the most abundant meioinvertebrates recorded in the surveyed shipment, there is no information about possible risk of their becoming invasive. These tiny organisms probably escape the attention of scientists due to their size and their problematic identification. Especially rotifers but to an extent also bryozoans might have high invasive potential inasmuch as they have the ability to disperse via wind or waterfowl and their sexual/asexual breeding system is such that even one egg can lead to successful colonization^27^. In contrast to the numerous studies about the effects of invasive aquatic plants on native biota^28^,^29^, there is a lack of information regarding associated zooplanktonic species. We assume, however, that they have a potential negative influence because certain other planktonic species have been assigned as invasive. These include, for example, the cladocerans *Bythotrephes longimanus* (Leydig) and *Daphnia lumholtzi* Sars, with demonstrated negative effects^30^,^31^, and the copepods *Acartia tonsa* Dana and *Eurytemora americana* Williams, with as yet unclear effects, on local communities^32^.

The most abundant group of captured macroinvertebrates comprises at least three species of freshwater snails (Table 1). In Indonesia, some species of freshwater snails have been reported as the intermediate hosts of helminths^33^. This case demonstrates three trophic levels which may be carried simultaneously. Moreover, there are some other related freshwater snail species introduced via the pet trade and recorded as invasive in Europe^34^,^35^,^36^,^37^. In addition, the import to and spreading the import to and spreading of snails belonging to the genus *Pomacea* within the European Union (EU) have been totally banned of snails belonging to the genus *Pomacea* within the European Union (EU) have been totally banned by the European Commission (Commission Implementing Decision 2012/697/EU of 8 November 2012), but no restrictions have focused especially on incidentally transported gastropods. It follows that the situation should be seen as cause for alarm because the aforementioned snails are released together with water hyacinths directly into garden ponds and hence their possible spread to new locations is both likely and apparently effortless.

The species with probably highest invasive potential of all of those macroinvertebrates found is the Indo–Australian moth *Spodoptera litura* (Fabricius). This moth is relatively tolerant of lower temperatures of about 10 °C^37^ and therefore has the potential to adapt to new climatic and ecological conditions. Although this species had not been recorded in the Czech Republic, it had previously been described in other European countries, including Denmark^39^, France^40^, Germany, the Netherlands, Russia^41^, and the UK^42^. Most of these introductions were successfully eradicated, but previous reproduction and grazing had been observed on various plant hosts and causing severe damage^42^. This moth is polyphagous and has been reported to feed on 112 cultivated food plant species within 44 families from across the world^43^. In case of its eventual successful establishment and spread, it has the potential to negatively impact many cultivated crops such as maize, tomato, cauliflower, and clover.

Precise evidence regarding the quantities of water hyacinths imported into Europe is not known. Rough estimates range from tens to hundreds of thousands of plants annually (V. Hořánek, pers. comm.). Although shipments of aquatic plants are sorted and subsequently inspected by authorized phytosanitary administrations in Indonesia as well as in the Czech Republic, many meio- and macroinvertebrates were imported in the surveyed plants.

Water hyacinths are usually placed into garden ponds situated outdoors. Associated animals, which may become established at a new location, have to survive the physical and chemical conditions of the receiving water but need not overcome physical barriers as do animals which are kept within indoor tanks or greenhouses^42^. The Czech Republic is a hub for imports of ornamental aquatic organisms into the EU from third countries^2^,^44^,^45^, and the further distribution of unwanted “hitchhiker” species throughout EU is easy due to the free movement of goods.

Although it is assumed that many tropical invertebrates are unable to overwinter in the temperate zone, some exceptions exist in thermal waters of either natural or anthropogenic origin involving fish^46^, shrimps^47^, and crayfish^48^. Although it is obvious that this type of environment is limited, ongoing climate changes may bring new possibilities of survival at locations where it would not be expected at present^49^. In addition, unwanted “hitchhikers” may serve as transmitters of various pathogens^50^.

The present study is not a complete list of animals associated with traded water hyacinths, and we are doubtful that such a list can ever be fully complete. Nevertheless, our study does bring a conclusive answer to the stated research question. The transport of water hyacinths within the ornamental trade is in fact associated with imports of unwanted “hitchhikers” which are potentially invasive species.

Although our findings were obtained in the Czech Republic, it is most probable that similar situations exist also in other countries. Therefore, the following management suggestions may minimize the risks of accidental introduction of invertebrates associated with water hyacinths: (1) detailed monitoring of imported shipments with a focus on macroinvertebrates, and (2) mandatory disinfection of imported water hyacinths by soaking in a substance such as potassium permanganate for 10 min to kill “hitchhiking” faunal assemblages.

## Methods

The examined water hyacinths had been farmed in outdoor ponds in Java, Indonesia, which is the main supplier of these plants to Europe. The plants were packed on 23 May 2015, and the air shipment arrived in the Czech Republic on 26 May as a regular commercial shipment. Water hyacinths were shipped unaccompanied by other goods in insulated polystyrene cartons without standing water and on a moist medium of wet newspapers inside polyethylene sacks. The shippers were independent of our team and did not know that the shipment would be inspected. Therefore, the examined shipment should be considered a “typical” shipment from Indonesia. On the arrival day, we surveyed in detail one box of water hyacinths (216 plants) immediately after unpacking.

### Data collection

When the shipment was received, the sacks were inspected for how much and which species had fallen off, or become dislodged, from the plants. Individual plants were visually examined and subsequently rinsed using a wash bottle (250 ml, Vitrum Praha, Czech Republic) with sterilized water. Macroinvertebrates were collected with forceps and kept in labelled 30 ml sample bottles and immediately fixed in 70% ethanol. The rinse water containing the organic matter and meioinvertebrates (including protists) was subsequently filtered through a polyamide sieve (42 μm mesh dimension, Silk and Progress, Czech Republic). The sieve was thoroughly washed between samples; all captured organic matter and organisms were held in labelled 20 ml sample bottles and immediately fixed in 70% ethanol. Prior to identification, a suspension of 1.5 ml from each 20 ml sample bottle was transferred to a Sedgewick-Rafter counting cell (Pyser-SGI, UK). Subsequently, all organisms were identified and counted using an Olympus BX51 binocular microscope. Macroinvertebrates were counted separately.

### Species determination

Both meio- and macroinvertebrate samples were identified to the lowest possible taxonomic unit. Various determination keys^51^,^52^,^53^ were used. The examined hyacinths were subsequently incubated over a period of 3 weeks at 23 °C in a 16:8 h light:dark cycle in sterilized water. After this period, all plants as well as the water in which they had been kept were once again examined in the same manner as described above for the presence of potentially hatched animals. The recorded invertebrate species are presented as the total number of individuals found in the shipment as well as the relative abundance per plant.

### Statistical analysis

For comparison of influence of rinsing and incubation on presence of “hitchhikers” we used Wilcoxon paired test and McNemar’s test. Wilcoxon paired test was used for detection of differences in numbers of individuals from each taxonomic group before and after incubation. McNemar’s test was used for comparison of presence or absence of taxonomic group of hitchhikers before and after incubation. Non-parametric Wilcoxon test was used due to non-normal character of data. The kind of data was based on a subsample (n = 8).

## Additional Information

**How to cite this article**: Patoka, J. *et al.* Potential pest transfer mediated by international ornamental plant trade. *Sci. Rep.* **6**, 25896; doi: 10.1038/srep25896 (2016).

## Figures and Tables

**Table 1:** Total numbers of meio- and macroinvertebrate taxa found on water hyacinths (*Eichhornia crassipes*) imported from Indonesia into the Czech Republic via the ornamental plant trade and their relative abundance.

Major group	Subordinate group	Taxon	Total number [ind.]	Relative abundance per plant [%]
Tubulinea	Arcellidae	*Arcella* sp.	3,667	11.21
	Centropyxidae	*Centropyxis* sp.	3,187	9.74
	Difflugiidae	u	1,193	3.65
Rotifera	Monogononta	*Lecane bulla*	1,547	4.73
		*Lecane leontina*	894	2.73
		*Lecane stichaea*	253	0.77
		u	387	1.18
	Bdelloidea	u	1,093	3.34
Tricladida	u	u	93	0.28
Bryozoa	u	u	547*	1.67
Nematoda	Tylenchida	u	853	2.61
Gastropoda	Planorbidae	*Gyraulus* sp.	108	0.33
	Lymnaeidae	*Radix* sp.	1	3.1 × 10^−3^
	Viviparidae	u	24	0.07
Annelida	Oligochaeta	u	748	2.29
Acari	Hydracarina	u	80	0.24
		u	67	0.20
Cladocera	Chydoridae	*Oxyurella* sp.	53	0.16
Copepoda	Cyclopidae	*Microcyclops* sp.	120	0.37
	Harpacticoida	u	53	0.16
Ostracoda	u	u	1	3.1 × 10^−3^
Collembola	u	u	53	0.16
Odonata	Anisoptera	u	14	0.04
	Zygoptera	u	1	3.1 × 10^−3^
Heteroptera	Naucoridae	*Ilyocoris* sp.	1	3.1 × 10^−3^
	Nepidae	*Ranatra* sp.	4	0.01
	u	u	1	3.1 × 10^−3^
Diptera	Psychodidae	*Psychoda* sp.	3	0.01
	Chironomidae	u	13	0.04
	Ceratopogonidae	u	2	0.01
Hymenoptera	Vespoidea	u	1	3.1 × 10^−3^
	Chalcidoidea	u	13	0.04
Thysanoptera	Thripidae	u	13	0.04
Coleoptera	Hydrophilidae	u	29	0.09
	Dytiscidae	u	14	0.04
	Curculionidae	u	13	0.04
	Elmidae	u	1	3.1 × 10^−3^
Lepidoptera	Noctuidae	*Spodoptera litura*	1	3.1 × 10^−3^
	u	u	1	3.1 × 10^−3^

All identified species are native to Asia. u = unknown (exact identification was ambiguous).

^*^Statoblasts only.
